# Association between adjuvant chemotherapy and survival in patients with rectal cancer and pathological complete response after neoadjuvant chemoradiotherapy and resection

**DOI:** 10.1038/s41416-020-0989-1

**Published:** 2020-07-29

**Authors:** Fang He, Huai-Qiang Ju, Yi Ding, Zhiqiang Jiang, Zhenhui Li, Bo Huang, Xiuhong Wang, Yuanyuan Zhao, Yong Li, Bin Qi, Wenguang Luo, Zijian Zhang, Qian Pei, Haiyang Chen, Shuai Liu, Xiaolin Pang, Jian Zheng, Jianping Wang, Jaffer A. Ajani, Xiang-Bo Wan

**Affiliations:** 1 Department of Radiation Oncology, The Sixth Affiliated Hospital of Sun Yat-sen University, Guangzhou, China; 2 Collaborative Innovation Center for Cancer Medicine, State Key Laboratory of Oncology in South Chin, Sun Yat-sen University Cancer Center, Guangzhou, China; 3 Department of Radiation Oncology, Nanfang Hospital of Southern Medical University, Guangzhou, China; 4 Department of General Surgery, Henan Cancer Hospital, Affiliated Tumor Hospital of Zhengzhou University, Zhengzhou, China; 5 Department of Radiology, The Third Affiliated Hospital of Kunming Medical University, Yunnan Cancer Hospital, Yunnan Cancer Center, Kunming, China; 6 Central Laboratory, Department of Pathology, Liaoning Cancer Hospital and Institute, Cancer Hospital of China Medical University, Liaoning, China; 7 Department of Pathology, China-Japan Friendship Hospital, Beijing, China; 8 Department of Radiation Oncology, The Affiliated Hospital of Qingdao University Medical College, Qingdao, China; 9 Department of General Surgery, Guangdong Provincial People’s Hospital, Guangdong Academy of Medical Sciences, Guangzhou, China; 10 Department of Radiation Oncology, Affiliated Cancer Hospital and Institute of Guangzhou Medical University, Guangzhou, China; 11 Department of Radiation Oncology, The First Affiliated Hospital of University of Science and Technology of China, Hefei, China; 12 Department of Radiation Oncology, Xiangya Hospital Central South University, Changsha, China; 13 Department of General Surgery, Xiangya Hospital Central South University, Changsha, China; 14 Department of Colorectal Surgery, The Sixth Affiliated Hospital of Sun Yat-sen University, Guangzhou, China; 15 Department of Gastrointestinal Medical Oncology, The University of Texas MD Anderson Cancer Center, Houston, TX USA

**Keywords:** Chemotherapy, Rectal cancer

## Abstract

**Background:**

For patients with locally advanced rectal cancer (LARC), it is unclear whether neoadjuvant chemoradiotherapy-induced pathologic complete response (pCR) individuals would further benefit from adjuvant chemotherapy (ACT).

**Methods:**

The pCR individuals who received different ACT cycles were paired by propensity score matching. Overall survival (OS), disease-free survival (DFS), local recurrence-free survival (LRFS), and distant metastasis-free survival (DMFS) were calculated by Kaplan–Meier and log-rank test.

**Results:**

In total, 1041 pCR individuals were identified from 5567 LARC cases. Specifically, 303 pCR cases had no ACT treatment, and 738 pCR patients received fluoropyrimidine-based ACT (median, 4 cycles) treatment. After 1:3 propensity score matching, 297 cases without ACT treatment were matched to 712 cases who received ACT treatment. Kaplan–Meier analysis showed that pCR individuals treated with or without ACT had the similar 3-year outcome (OS, DFS, LRFS and DMFS) (all *P* > 0.05). Moreover, the pCR patients received different ACT cycle(s) (0 vs. 1–4 cycles, 0 vs. ≥5 cycles) had comparable 3-year OS, DFS, LRFS and DMFS (all *P* > 0.05). In stratified analysis, ACT treatment did not improve 3-year survival (OS, DFS, LRFS and DMFS) for the baseline high-risk (cT3–4/cN1–2) subgroup patients (all *P* > 0.05).

**Conclusion:**

ACT, which did not improve survival, is unnecessary to neoadjuvant treatment-induced pCR LARC patients.

**Trial registration:**

2019ZSLYEC-136 (24-6-2019).

## Background

Currently, the standard therapeutic regimen for locally advanced rectal cancer (LARC) is neoadjuvant chemoradiotherapy (nCRT), followed by total mesorectal excision (TME) and adjuvant chemotherapy (ACT).^1–3^ The majority of LARC patients achieve tumour downsizing and downstaging after nCRT,^4–6^ and 15–38% of these patients would have a pathologic complete response (pCR, defined as ypT0N0),^7–9^ which is associated with an excellent long-term survival outcome.^5,10^

The administration of ACT to patients with LARC has been challenging, since the survival benefits of ACT were extrapolated from studies of colon cancer.^11–13^ Although National Comprehensive Cancer Network (NCCN) guideline recommend ACT after nCRT and TME, the role of ACT in the treatment of LARC remains unclearly. The large size, prospective QUASAR study reported that ACT with fluorouracil and folinic acid could improve survival for patients with stage II colorectal cancer, although the absolute improvements were small.^14^ The European Organisation for Research and Treatment of Cancer (EORTC) trial 22921, the Dutch PROCTOR/SCRIPT trial and the I-CNR-RT trial all reported that ACT conferred no overall survival (OS) or disease-free survival (DFS) benefit.^15–17^ However, these trials were underpowered to detect survival benefits due to poor accrual or compliance. Thus far, available reported data do not significantly support the routine administration of ACT for LARC patients treated with nCRT and TME surgery.^18^

Given the favourable prognosis of the pCR subgroup LARC patients,^5,10^ the rational of adding ACT has been questioned again.^19^ The National Cancer Database (NCDB)-derived analysis reported that ACT might prolong OS for the pCR subgroup of patients with LARC.^14,20**–**22^ For example, a survival analysis of 2455 pCR patients screened from the NCDB detected a 3.6% OS benefit (with ACT vs. without ACT: 97.6% vs. 94.0%), particularly in patients with baseline node-positive disease.^23^ Moreover, several NCDB data-based meta-analysis indicated that ACT was associated with improved OS in LARC patients with pCR after nCRT and TME surgery.^24,25^ In contrast, other meta-analyses and multicentre-based studies found that, compared to non-ACT treatment, ACT did not improve the OS in the pCR subset of patients with LARC.^26^ A long-term analysis of 566 pCR patients from the Gastro-Intestinal Working Group of the Italian Association of Radiation Oncology database found that ACT was even associated with a worse outcome (borderline significance).^27^ Therefore, the efficacy of ACT remains controversial in patients who achieve pCR after nCRT.^28^

The objective of this study was to assess whether ACT treatment would have any survival outcome benefit in LARC patients who achieved pCR after nCRT treatment and TME surgery.

## Methods

### Study population

This study recruited LARC patients (clinically T3–T4 and/or N positive) with tumours within 15 cm of the anal verge from January 2010 to December 2018. All cases were diagnosed by colonoscopy biopsy and histologic examination. The clinical tumour-node-metastasis (TNM) stage of each LARC patient was defined by contrast-enhanced whole-body computed tomography scan, transrectal ultrasound, or contrast-enhanced pelvic magnetic resonance imaging. After nCRT treatment and TME surgery, pathologically confirmed ypT0N0M0 patients were studied to detect the association between ACT and prognosis. Exclusion criteria were patients with R1 or R2 resection, given >7 cycles of nCRT, previous cancer history or with microscopic tumour cell appearance (ypT_0–4_N_1–2_ or ypT_1–4_N_0_). This study was approved by the Clinical Ethics Review Committee at the Sixth Affiliated Hospital of Sun Yat-sen University.

### Treatment

All patients received neoadjuvant treatment and TME surgery. Briefly, the neoadjuvant radiotherapy was delivered by direct-beam radiation of 50.4 Gy in 25 fractions and concurrently administered fluoropyrimidine, either orally or intravenously. To achieve substantial tumour downsizing and downstaging, some patients were treated with fluoropyrimidine-based chemotherapy before concurrent chemoradiotherapy. Also, during the 4–8 weeks waiting time before TME operation, some patients were given fluoropyrimidine-based consolidation chemotherapy. A group of patients with small LARC did not receive preoperative radiotherapy. The curative intent operation was performed according to TME principles 4–8 weeks after completion of neoadjuvant treatment. Fluoropyrimidine-based ACT was administered to most of patients, and the cycles of ACT to be given were at the physician’s discretion.

### Follow-up

After TME surgery, all patients were followed up at 3-month intervals during the first 3 years and at 6-month intervals thereafter, with physical examinations, contrast-enhanced pelvic magnetic resonance imaging, complete biochemistry and tumour biomarker tests. A contrast-enhanced whole-body computed tomography scan and a colonoscopy were performed annually. OS was defined as time from the date of diagnosis to death or, when censored, at the latest date if the patient was still alive. DFS was defined as time from the date of surgery to the date of disease relapse, the date of death or, when censored, at the latest time. Local recurrence-free service (LRFS) and distant metastasis-free survival (DMFS) were defined as time from diagnosis to the date of local or distant recurrence, respectively, or the date of death or, when censored, at the latest date.

### Propensity score matching

The propensity score model was used to minimise the potential bias caused by confounding covariates. A multivariable logistic regression model was constructed to generate propensity scores. The clinicopathologic factors included in the model were age (≤55 or >56 years) at diagnosis, sex, clinical stage (II or III), preoperative clinical T stage, clinical N stage, radiotherapy (with or without), histologic grade (high, moderate or poor differentiation), tumour distance from anus (≤5, 5–10 or >10 cm) and cycles of nCRT courses (0, ≤3 or 4–7 cycles). Patients who received ACT were matched to that who did not receive ACT at a 1:1, 1:2 and 1:3 ratio, respectively, using a greedy nearest-neighbour matching algorithm with no replacement. A calliper width equal to 0.2 of the standard deviation was used as the logit of the propensity score. Patient characteristics between the propensity score-matched groups were compared using *P* values.

### Statistical analysis

Kaplan–Meier survival curves were used to calculate OS, DFS, LRFS and DMFS ratios between the propensity score-matched subgroup patients. Statistical differences between curves were assessed using the log-rank test. All *P* values were two sided, and *P* values <0.05 were considered as statistically significant. Statistical analyses were performed with SPSS software, version 24.0 (SPSS Inc., Chicago, IL).

## Results

### Patient characteristics

**Fig. 1:**
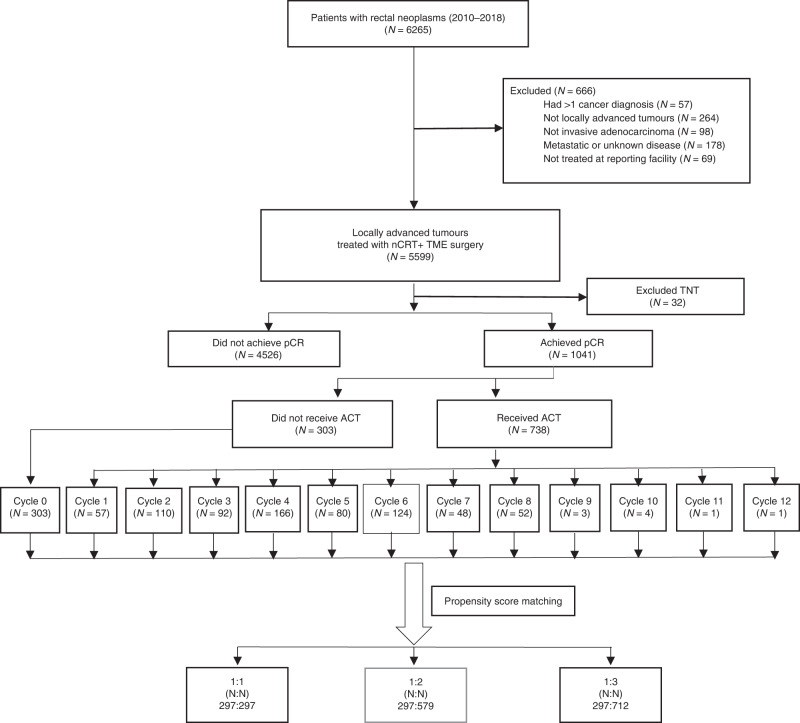
The Consort diagram.

Of 5567 consecutive nCRT and TME surgery-treated LARC cases, 1041 cases (18.7%) achieved a pCR (median age, 55.0 years; 56.9% of male). For the pCR subgroup patients, 303 (29.1%) cases did not receive ACT treatment. Seven hundred and thirty-eight (70.9%) patients received ACT (range, 1–12 cycles; median, 4 cycles) treatment. Of whom, 57.6% of individuals (425/738) received 1–4 cycles of ACT and 42.4% of patients (313/738) received 5 or more cycles of ACT treatment (Fig. 1). As shown in Table 1 and Supplementary Table 1, except for the number of ACT cycles (*P* < 0.001) and tumour location (*P* < 0.05), all other patient characteristics were similar between the subgroup patients before and after propensity score matching (all *P* > 0.05) (Supplementary Table 2).

**Table 1:** Patients baseline characteristics before and after propensity score matching.

Characteristics	Before matching			After matching (1:3)		
	ACT no. (%) (*n* = 738)	No ACT no. (%) (*n* = 303)	*P* value	ACT no. (%) (*n* = 712)	No ACT no. (%) (*n* = 297)	*P* value
Age, median 55 years						
≤55	388 (52.6)	147 (48.5)	0.234	370 (52.0)	147 (49.5)	0.474
>55	350 (47.4)	156 (51.5)		342 (48.0)	150 (50.5)	
Gender						
Male	418 (70.6)	174 (29.4)	0.763	454 (63.8)	191 (64.3)	0.869
Female	224 (71.6)	89 (28.4)		258 (36.2)	106 (35.7)	
Clinical T stage						
cT1	1 (0.1)	0 (0.0)	0.893	1 (0.1)	0 (0.0)	0.922
cT2	36 (4.9)	13 (4.3)		34 (4.8)	12 (4.0)	
cT3	462 (62.6)	196 (64.7)		449 (63.1)	194 (65.3)	
cT4	239 (32.4)	94 (31.0)		228 (32.0)	91 (30.7)	
Clinical N stage						
cN0	160 (21.7)	74 (24.4)	0.442	156 (21.9)	73 (24.6)	0.647
cN1	522 (70.7)	189 (62.4)		501 (70.4)	188 (63.3)	
cN2	56 (7.6)	40 (13.2)		55 (7.7)	36 (12.1)	
Clinical stage						
II	172 (23.3)	77 (25.4)	0.469	156 (21.9)	73 (24.6)	0.357
III	566 (76.7)	226 (74.6)		556 (78.1)	224 (75.4)	
Location from anal verge (cm)						
0–5	396 (53.7)	197 (65.0)	0.002	393 (55.2)	191 (64.3)	0.016
5–10	319 (43.2)	97 (32.0)		296 (41.6)	97 (32.7)	
>10	23 (3.1)	9 (3.0)		23 (3.2)	9 (3.0)	
Tumour differentiation						
Highly differentiated	135 (18.2)	56 (18.5)	0.550	130 (18.3)	56 (18.9)	0.716
Moderately differentiated	472 (64.0)	200 (66.0)		463 (65.0)	194 (65.3)	
Poorly differentiated	131 (17.8)	47 (15.5)		119 (16.7)	47 (15.8)	
Radiation or not						
Radiation	653 (88.5)	271 (89.4)	0.657	629 (88.3)	265 (89.2)	0.688
No radiation	85 (11.5)	32 (10.6)		83 (11.7)	32 (10.8)	
NCT cycle, median 3 cycles						
0	4 (0.6)	4 (1.3)	0.346	4 (0.6)	2 (0.7)	0.595
1–3	367 (49.7)	156 (51.5)		355 (49.9)	153 (51.5)	
4–7	367 (49.7)	143 (47.2)		353 (49.5)	142 (47.8)	
ACT cycle, median 4 cycles						
0	0 (0.0)	303 (100.0)	<0.001	0 (0.0)	297 (100.0)	<0.001
1–4	425 (57.6)	0 (0.0)		403 (56.6)	0 (0.0)	
≥5	313 (42.4)	0 (0.0)		309 (43.4)	0 (0.0)	

*NCT* neoadjuvant chemotherapy, *ACT* adjuvant chemotherapy.

### Propensity score matching

Here, the propensity score model matched the clinicopathologic variables between or among different subgroup patients, including age (≤55 or >56 years), sex, clinical TNM stage, clinical T stage, clinical N stage, radiotherapy (with or without), histologic grade (high, moderate or poor differentiation), tumour distance from anus (≤5, 5–10 or >10 cm) and nCRT cycles (0, 1–3 or 4–7 cycles). In total, 712 patients who received ACT treatment were matched to 297 patients who did not receive ACT (1:3 matching) treatment. After propensity score matching, the standardised differences of included covariates between these two subgroups were all <0.1 (Supplementary Fig. 1), suggesting a well-balanced covariate distribution of these two subgroup patients. We also investigated the correlation between ACT and survival outcome at 1:1 and 1:2 propensity score matching. As shown in Supplementary Table 1, the included covariates between two subgroups were all similar, at 1:1 or at 1:2 matching (Supplementary Fig. 2).

### Association of ACT treatment and survival outcome

**Fig. 2:**
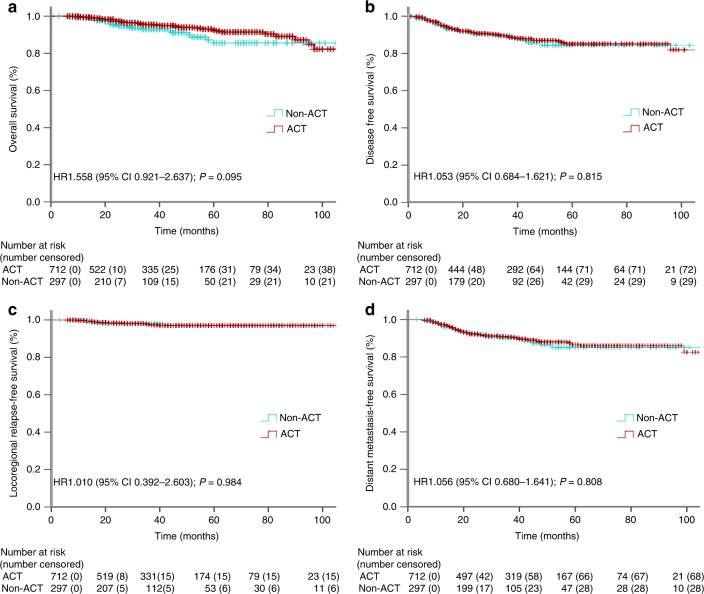
The Kaplan–Meier curve analysis of OS (**a**), DFS (**b**), LRFS (**c**) and DMFS (**d**) for pCR LARC patients treated with or without ACT after propensity score matching (1:3).

The median follow-up time for the entire cohort was 35.0 months (interquartile range, 19.0–57.5 months). At 1:3 matching, the ACT and non-ACT subgroup patients had similar OS ratios (Fig. 2a): the 3-year OS rate was 95.5% for the ACT subgroup and 93.0% for the non-ACT subset (*P* = 0.095; hazard ratio [HR], 1.558; 95% confidence interval [CI], 0.921–2.637). Also, these two subgroups had a similar DFS rates (Fig. 2b): the 3-year DFS rate was 89.2% for the ACT subgroup and 89.6% for the non-ACT subset (*P* = 0.815; HR, 1.053; 95% CI, 0.684–1.621). Moreover, a comparable LRFS (Fig. 2c) and DMFS (Fig. 2d) rates were also noted in these two subgroups: the 3-year LRFS rate was 97.6% for the ACT subgroup and 98.0% for the non-ACT subset (*P* = 0.984; HR, 1.010; 95% CI, 0.392–2.603), and the 3-year DMFS rate was 90.7% for the ACT subset and 90.1% for the non-ACT subset (*P* = 0.808; HR, 1.056; 95% CI, 0.680–1.641). Additionally, at 1:1 and 1:2 matching, similar survival (OS, DFS, LRFS and DMFS) ratios between these two subgroup patients were also observed (all *P* > 0.05; Supplementary Figs. 3 and 4).

**Fig. 3:**
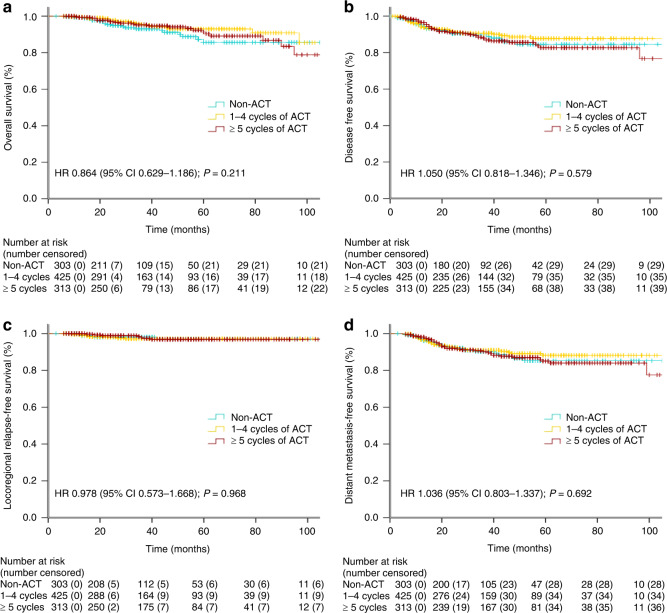
The Kaplan–Meier curve analysis of OS (**a**), DFS (**b**), LRFS (**c**) and DMFS (**d**) for pCR LARC patients who did not receive ACT, who received 1–4 cycles of ACT and who received 5 or more cycles of ACT.

Next, we investigated whether different ACT cycles affected survival outcomes for pCR LARC patients. As shown in Fig. 3, the subgroups received 0 (303 patients), 1–4 (425 patients) and 5 or more cycles (313 patients) of ACT treatment had similar 3-year OS, DFS, LRFS and DMFS rates (all *P* > 0.05).

Similarly, the propensity score model was also performed by matching the clinicopathologic factors of age (≤55 or >56 years), sex, clinical TNM stage, clinical T stage, clinical N stage, radiotherapy (with or without), histologic grade (high, moderate or poor differentiation), tumour distance from anus (≤5, 5–10 or >10 cm) and nCRT cycles (0, 1–3 or 4–7 cycles). At 1:3 matching, two subgroup patients were identified according to their ACT cycles: 294 pCR patients who did not receive ACT and 422 patients who received 1–4 cycles of ACT. Similarly, we also matched 276 pCR patients who did not receive ACT to 311 patients who received 5 or more cycles of ACT. Compared to observation (0 cycle), 1–4 cycles of ACT treatment conferred no outcomes (OS, DFS, LRFS and DMFS) benefit to pCR patients (all *P* > 0.05, Supplementary Fig. 5). As expected, observation (0 cycle) and ≥5 cycles of ACT treatment was correlated with similar 3-year OS, DFS, LRFS and DMFS ratios (all *P* > 0.05, Supplementary Fig. 6).

### Subgroup analysis (baseline clinical T3–4 and/or N-positive stage subgroups)

**Fig. 4:**
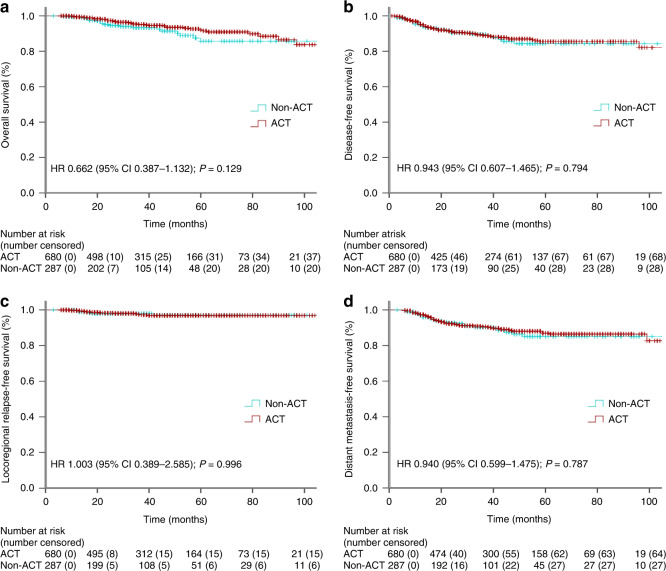
The Kaplan–Meier curve analysis of OS (**a**), DFS (**b**), LRFS (**c**) and DMFS (**d**) for pCR LARC patients, who had baseline cT3–4 stage, treated with or without ACT (1:3 matching).

**Fig. 5:**
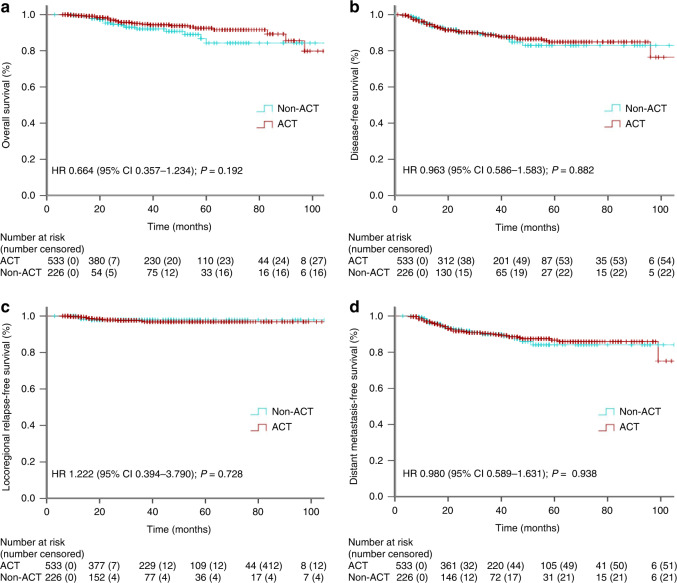
The Kaplan–Meier curve analysis of OS (**a**), DFS (**b**), LRFS (**c**) and DMFS (**d**) for pCR patients, who had baseline cN-positive stage, treated with or without ACT (1:3 matching).

Previous studies reported that pCR patients who were of baseline cT3–4 or cN-positive stage might benefit most from ACT treatment.^23,29^ Here, using propensity score matching method, we identified 967 cT3–4 stage patients who received (680 patients) or did not receive (287 patients) ACT treatment, by matching the clinicopathologic factors of age (≤55 or >56 years), sex, clinical TNM stage, clinical N stage, radiotherapy (with or without), histologic grade (high, moderate or poor differentiation), tumour distance from anus (≤5, 5–10 or >10 cm), and nCRT cycles (0, 1–3 or 4–7 cycles). The survival analysis confirmed that ACT confers no survival outcomes (OS, DFS, LRFS and DMFS) benefit to baseline cT3–4 stage pCR LARC patients (all *P* > 0.05; Fig. 4). Moreover, the matched 759 cN-positive cases (with vs. without ACT treatment: 533 vs. 226 patients) were also subjected to the Kaplan–Meier survival analysis. Similarly, ACT treatment did not correlate with statistically improved outcomes (OS, DFS, LRFS and DMFS) for baseline cN-positive stage pCR LARC patients (all *P* > 0.05; Fig. 5).

## Discussion

The standard regimen for patients with stage cT3–4 or cN-positive LARC is nCRT, TME surgery and 6 months of perioperative chemotherapy.^1^ However, the lack of direct evidence to support the use of ACT made the indication for ACT is questionable in LARC, particularly in nCRT-induced pCR patients who have favourable long-term outcome.^30–32^ In this study, a consecutive cohort of 1041 pCR patients was enrolled to evaluate the association between ACT treatment and outcome. We confirmed that ACT did not improve the long-term outcomes for LARC patients who have achieved pCR, even if given intensified ACT cycles. Importantly, once the baseline high-risk patients achieve pCR after the nCRT, ACT treatment would confer no additional survival benefit.

Agreeing with our study, several randomised phase 3 trials studied the effect of ACT for LARC, and none proved a survival benefit from ACT treatment. The four-arm randomised EORTC 22921 trial examined the subgroups of patients in nCRT vs. neoadjuvant radiotherapy alone and ACT vs. observation in LARC.^15^ Compared to the observational arm, the 10-year long-term OS and DFS of other three arms were similar. Subsequently, two randomised clinical trials, the Dutch PROCTOR-SCRIPT trial and the I-CNR-RT trial, investigated the survival benefit of ACT for LARC.^16,17^ These three trials all showed that, compared to observation alone, ACT did not improve OS and DFS for LARC patients, including the subgroup of pCR patients. However, their conclusions were questioned, since poor compliance to ACT protocol or premature closure due to poor accrual limited their power to detect survival benefit. Thus far, available data do not robustly support the routine use of ACT for LARC patients treated with nCRT and TME surgery.^18^

In clinical practice, European Society for Medical Oncology (ESMO) guideline recommends the ACT only to ypIII stage or high-risk ypII stage LARC patients, rather than to pCR individuals.^33^ Similarly, although NCCN guideline does not give a clear treatment recommendation to pCR individuals, an observation, but not ACT, is recommended for ypT1-2N0M0 patients.^1^ Therefore, it is reasonable to believe that the option of observation should be an appropriate choice for the pCR individuals, who theoretically have a favourable survival outcome equal to or better than ypT1-2N0M0 patients.^5^ Our findings were in line with these clinical guidelines, and found that ACT was unnecessary for pCR individuals. By contrast, several back-to-back observational cohort studies and meta-analysis were conducted that used the NCDB to investigate the association between ACT and OS for pCR subgroups of LARC patients.^20–25,29^ These studies concluded that ACT might improve OS. In these studies, ACT-treated pCR subgroups of patients, even among high-risk patients, accounted for 25.0–28.0% of the entire pCR patient cohort, reflecting that observation, but not given ACT treatment, was a more acceptable selection for physician and pCR individuals in North Americans. Moreover, their stratified analysis found that ACT was more likely to be given to younger patients (age <60 years) and to individuals with better performance status.^20^ It has been known that younger age and better performance status are the favourable and independent prognostic factors for OS, indicating that the OS benefit might come from younger age and better performance status rather than ACT treatment. Moreover, even the pCR subgroups patients were identified from the same database (NCDB, 24418–27879 LARC cases) at the same time period (2006–2012) for the same aim to detect the association of ACT with survival, the pCR patient numbers (2455 vs. 5606) and ratios (9.18% vs. 23.0%) in different studies were varied significantly.^22,23,29^ Therefore, the bias in patient selection, younger age and better performance status would cause an overestimation on the effect of ACT for OS to the pCR LARC patients.^30^

In this study, we identified patients who achieved pCR from consecutively treated patients with LARC. Indeed, LARC patients who achieved pCR at the rate of 18.7% in this study was similar to most of reported studies,^34^ suggesting that our pCR subgroup patients were representative and therefore ideal for further analysis. Moreover, even before propensity score matching, the median age and other important clinicopathologic variables (clinical TNM stage, age, sex, histologic grade and nCRT cycles) between the subgroup patients with and without ACT treatment were similar in this study (all *P* > 0.05), indicating that the clinicopathological features of enrolled pCR patients were balanced between these two subgroup patients. In NCDB and other registered databases, the ACT regimen and cycles, disease relapse, and cancer-related death information were always not included.^30^ Therefore, it is difficult to conclude that the OS benefit was due to ACT treatment, since improvement of OS was mainly translated from reduced disease relapse and cancer-related death. Here, we included ACT treatment regimen and cycle data (Fig. 1), and detailed survival outcome (OS, DFS, LRFS and DMFS) information of each pCR patient. Through propensity score matching in a large cohort (1041 patients), we proved that ACT did not improve OS, DFS, LRFS and DMFS, even considering the influencing factors of ACT cycles and high-risk stage (cT3–4 and cN positive at baseline) (Figs. 4 and 5).

Our study had limitations. First, the ACT regimens were varied among patients (391 received XELOX, 47 received De Gramont, 211 received FOLFOX, 61 received Xeloda, 19 received FOLFOXIRI and 9 received FOLFIRI), although previous studies suggested that there was no survival difference when patients received fluoropyrimidine-based ACT. Second, the ACT cycles were different among individual pCR patients (range, 1–12 cycles; median, 4 cycles). We mitigated this limitation by including a large number of patients (1041) and propensity score matching the ACT cycles. Third, this was a retrospective cohort study. The imbalanced clinicopathological characteristics among subgroup patients might be of potential biases. We minimised this issue by recruiting pCR subgroups from consecutive nCRT-treated patients and by propensity score matching the important confounding factors (Supplementary Figs. 1–3, Table 1). Additionally, compared with NCDB-based studies, our study included a smaller patient size, which probably lack the power to detect OS difference. Finally, the median follow-up time for the entire cohort was 35.0 months in present study. Although this time should be enough to detect the 3-year survival outcome difference between subgroup patients, the findings of this study should be warranted by long-term follow-up and other prospective clinical trials.

## Conclusions

Our study demonstrated that ACT treatment was not associated with prolonged survival outcome in nCRT-induced pCR LARC patients, even if the intensified ACT cycles were given. Moreover, when the baseline high-risk LARC patients achieve pCR, ACT treatment would not confer additional survival benefit. Therefore, ACT is unnecessary to pCR LARC patients and should be omitted.

## Supplementary information

supplementary file

## Data Availability

The datasets used during the current study are available from the corresponding author on reasonable request.

## References

[1] NCCN. *NCCN Clinical Practice Guidelines in Oncology: Rectal Cancer* (version 2.2019). 10.6004/jnccn.2009.0057. 10.6004/jnccn.2009.0057 19755047

[2] Sauer R, Becker H, Hohenberger W, Rodel C, Wittekind C, Fietkau R (2004). Preoperative versus postoperative chemoradiotherapy for rectal cancer. N. Engl. J. Med..

[3] Sebag-Montefiore D, Stephens RJ, Steele R, Monson J, Grieve R, Khanna S (2009). Preoperative radiotherapy versus selective postoperative chemoradiotherapy in patients with rectal cancer (MRC CR07 and NCIC-CTG C016): a multicentre, randomised trial. Lancet.

[4] Collette L, Bosset JF, den Dulk M, Nguyen F, Mineur L, Maingon P (2007). Patients with curative resection of cT3–4 rectal cancer after preoperative radiotherapy or radiochemotherapy: does anybody benefit from adjuvant fluorouracil-based chemotherapy? A trial of the European Organisation for Research and Treatment of Cancer Radiation Oncology Group. J. Clin. Oncol..

[5] Maas M, Nelemans PJ, Valentini V, Das P, Rodel C, Kuo LJ (2010). Long-term outcome in patients with a pathological complete response after chemoradiation for rectal cancer: a pooled analysis of individual patient data. Lancet Oncol..

[6] Park IJ, You YN, Agarwal A, Skibber JM, Rodriguez-Bigas MA, Eng C (2012). Neoadjuvant treatment response as an early response indicator for patients with rectal cancer. J. Clin. Oncol..

[7] Garcia-Aguilar J, Chow OS, Smith DD, Marcet JE, Cataldo PA, Varma MG (2015). Effect of adding mFOLFOX6 after neoadjuvant chemoradiation in locally advanced rectal cancer: a multicentre, phase 2 trial. Lancet Oncol..

[8] Cercek A, Roxburgh CSD, Strombom P, Smith JJ, Temple LKF, Nash GM (2018). Adoption of Total Neoadjuvant Therapy for Locally Advanced Rectal Cancer. JAMA Oncol..

[9] Zhang JW, Cai Y, Xie XY, Hu HB, Ling JY, Wu ZH (2020). Nomogram for predicting pathological complete response and tumor downstaging in patients with locally advanced rectal cancer on the basis of a randomized clinical trial. Gastroenterol. Rep. (Oxf.).

[10] Fokas E, Strobel P, Fietkau R, Ghadimi M, Liersch T, Grabenbauer GG (2017). Tumor regression grading after preoperative chemoradiotherapy as a prognostic factor and individual-level surrogate for disease-free survival in rectal cancer.. J. Natl Cancer Inst.

[11] Moertel CG, Fleming TR, Macdonald JS, Haller DG, Laurie JA, Goodman PJ (1990). Levamisole and fluorouracil for adjuvant therapy of resected colon carcinoma. N. Engl. J. Med..

[12] Twelves C, Wong A, Nowacki MP, Abt M, Burris H, Carrato A (2005). Capecitabine as adjuvant treatment for stage III colon cancer. N. Engl. J. Med..

[13] Andre T, Boni C, Navarro M, Tabernero J, Hickish T, Topham C (2009). Improved overall survival with oxaliplatin, fluorouracil, and leucovorin as adjuvant treatment in stage II or III colon cancer in the MOSAIC trial. J. Clin. Oncol..

[14] Quasar Collaborative G, Gray R, Barnwell J, McConkey C, Hills RK, Williams NS (2007). Adjuvant chemotherapy versus observation in patients with colorectal cancer: a randomised study. Lancet.

[15] Bosset JF, Calais G, Mineur L, Maingon P, Stojanovic-Rundic S, Bensadoun RJ (2014). Fluorouracil-based adjuvant chemotherapy after preoperative chemoradiotherapy in rectal cancer: long-term results of the EORTC 22921 randomised study. Lancet Oncol..

[16] Sainato A, Cernusco Luna Nunzia V, Valentini V, De Paoli A, Maurizi ER, Lupattelli M (2014). No benefit of adjuvant Fluorouracil Leucovorin chemotherapy after neoadjuvant chemoradiotherapy in locally advanced cancer of the rectum (LARC): long term results of a randomized trial (I-CNR-RT). Radiother. Oncol..

[17] Breugom AJ, van Gijn W, Muller EW, Berglund A, van den Broek CB, Fokstuen T (2015). Adjuvant chemotherapy for rectal cancer patients treated with preoperative (chemo)radiotherapy and total mesorectal excision: a Dutch Colorectal Cancer Group (DCCG) randomized phase III trial. Ann. Oncol..

[18] Carvalho C, Glynne-Jones R (2017). Challenges behind proving efficacy of adjuvant chemotherapy after preoperative chemoradiation for rectal cancer. Lancet Oncol..

[19] Bujko K, Glynne-Jones R, Bujko M (2010). Does adjuvant fluoropyrimidine-based chemotherapy provide a benefit for patients with resected rectal cancer who have already received neoadjuvant radiochemotherapy? A systematic review of randomised trials. Ann. Oncol..

[20] Shahab D, Gabriel E, Attwood K, Ma WW, Francescutti V, Nurkin S (2017). Adjuvant chemotherapy is associated with improved overall survival in locally advanced rectal cancer after achievement of a pathologic complete response to chemoradiation. Clin. Colorectal Cancer.

[21] Xu Z, Mohile SG, Tejani MA, Becerra AZ, Probst CP, Aquina CT (2017). Poor compliance with adjuvant chemotherapy use associated with poorer survival in patients with rectal cancer: an NCDB analysis. Cancer.

[22] Turner MC, Keenan JE, Rushing CN, Gulack BC, Nussbaum DP, Benrashid E (2019). Adjuvant chemotherapy improves survival following resection of locally advanced rectal cancer with pathologic complete response. J. Gastrointest. Surg..

[23] Dossa F, Acuna SA, Rickles AS, Berho M, Wexner SD, Quereshy FA (2018). Association between adjuvant chemotherapy and overall survival in patients with rectal cancer and pathological complete response after neoadjuvant chemotherapy and resection. JAMA Oncol..

[24] Tomasello G, Ghidini M, Petrelli F (2019). Adjuvant chemotherapy in patients with rectal cancer achieving pathologic complete response after neoadjuvant chemoradiation and surgery. Eur. J. Cancer.

[25] Ma B, Ren Y, Chen Y, Lian B, Jiang P, Li Y (2019). Is adjuvant chemotherapy necessary for locally advanced rectal cancer patients with pathological complete response after neoadjuvant chemoradiotherapy and radical surgery? A systematic review and meta-analysis. Int. J. Colorectal Dis..

[26] Breugom AJ, Swets M, Bosset JF, Collette L, Sainato A, Cionini L (2015). Adjuvant chemotherapy after preoperative (chemo)radiotherapy and surgery for patients with rectal cancer: a systematic review and meta-analysis of individual patient data. Lancet Oncol..

[27] Capirci C, Valentini V, Cionini L, De Paoli A, Rodel C, Glynne-Jones R (2008). Prognostic value of pathologic complete response after neoadjuvant therapy in locally advanced rectal cancer: long-term analysis of 566 ypCR patients. Int. J. Radiat. Oncol. Biol. Phys..

[28] Beets GL, Glimelius BL (2014). Adjuvant chemotherapy for rectal cancer still controversial. Lancet Oncol..

[29] Polanco PM, Mokdad AA, Zhu H, Choti MA, Huerta S (2018). Association of adjuvant chemotherapy with overall survival in patients with rectal cancer and pathologic complete response following neoadjuvant chemotherapy and resection. JAMA Oncol..

[30] Chang GJ (2018). Is there validity in propensity score-matched estimates of adjuvant chemotherapy effects for patients with rectal cancer?. JAMA Oncol..

[31] Fokas E, Liersch T, Fietkau R, Hohenberger W, Beissbarth T, Hess C (2014). Tumor regression grading after preoperative chemoradiotherapy for locally advanced rectal carcinoma revisited: updated results of the CAO/ARO/AIO-94 trial. J. Clin. Oncol..

[32] Rodel C, Martus P, Papadoupolos T, Fuzesi L, Klimpfinger M, Fietkau R (2005). Prognostic significance of tumor regression after preoperative chemoradiotherapy for rectal cancer. J. Clin. Oncol..

[33] Glynne-Jones R, Wyrwicz L, Tiret E, Brown G, Rodel C, Cervantes A (2018). Rectal cancer: ESMO Clinical Practice Guidelines for diagnosis, treatment and follow-up. Ann. Oncol..

[34] Rödel C, Graeven U, Fietkau R, Hohenberger W, Hothorn T, Arnold D (2015). Oxaliplatin added to fluorouracil-based preoperative chemoradiotherapy and postoperative chemotherapy of locally advanced rectal cancer (the German CAO/ARO/AIO-04 study): final results of the multicentre, open-label, randomised, phase 3 trial. Lancet Oncol..

