# Supplementary material for: Association between adjuvant chemotherapy and survival in patients with rectal cancer and pathological complete response after neoadjuvant chemoradiotherapy and resection
Source: Br J Cancer. 2020 Jul 29;123(8):1244–52. doi: 10.1038/s41416-020-0989-1 (PMC7553967; doi:10.1038/s41416-020-0989-1)
Supplement: Supplementary file 1 — supplementary file [file 41416_2020_989_MOESM1_ESM.pptx]

## Slide 1
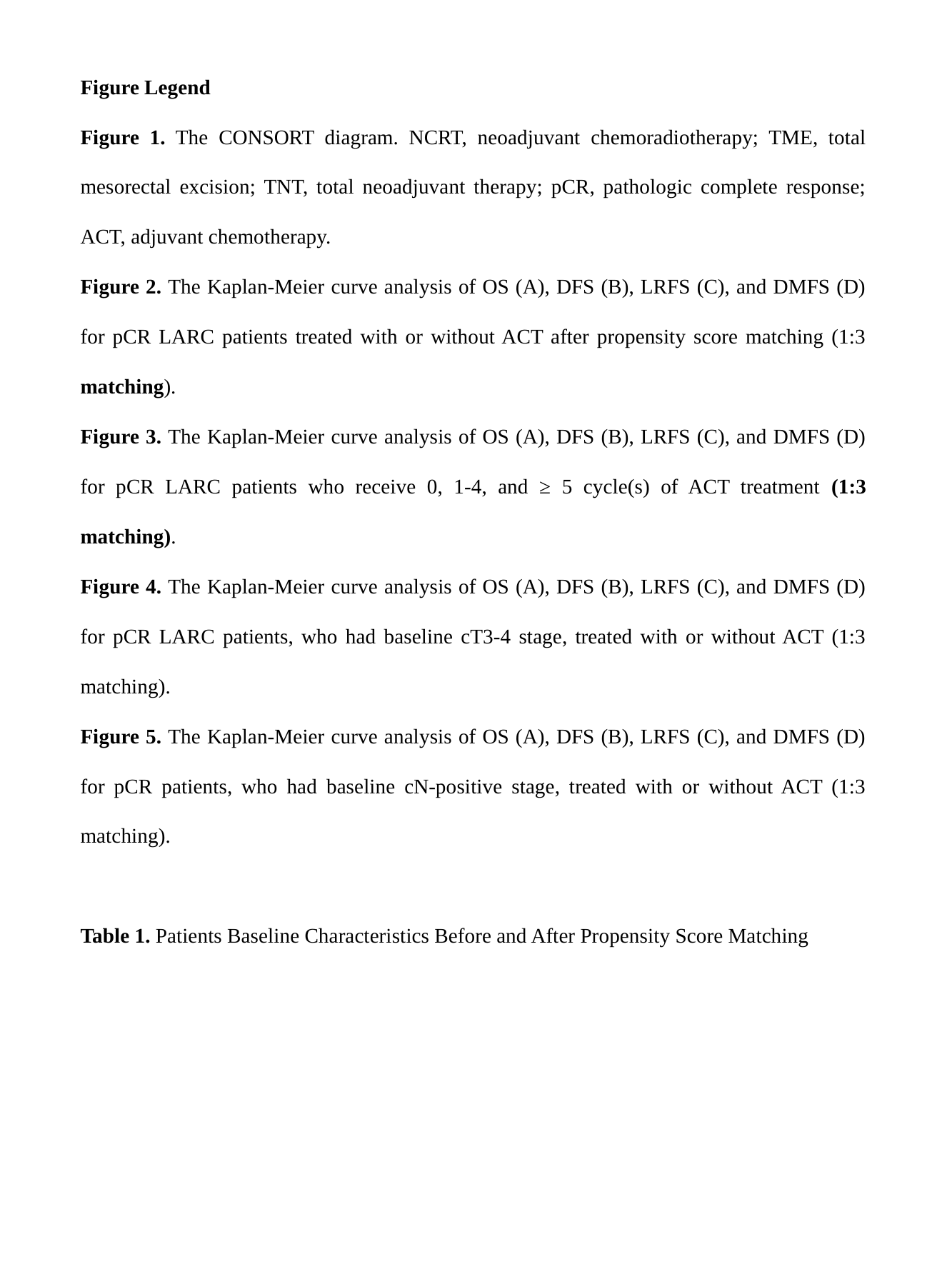

Figure Legend
Figure 1. The CONSORT diagram. NCRT, neoadjuvant chemoradiotherapy; TME, total mesorectal excision; TNT, total neoadjuvant therapy; pCR, pathologic complete response; ACT, adjuvant chemotherapy.
Figure 2. The Kaplan-Meier curve analysis of OS (A), DFS (B), LRFS (C), and DMFS (D) for pCR LARC patients treated with or without ACT after propensity score matching (1:3 matching).
Figure 3. The Kaplan-Meier curve analysis of OS (A), DFS (B), LRFS (C), and DMFS (D) for pCR LARC patients who receive 0, 1-4, and ≥ 5 cycle(s) of ACT treatment (1:3 matching).
Figure 4. The Kaplan-Meier curve analysis of OS (A), DFS (B), LRFS (C), and DMFS (D) for pCR LARC patients, who had baseline cT3-4 stage, treated with or without ACT (1:3 matching).
Figure 5. The Kaplan-Meier curve analysis of OS (A), DFS (B), LRFS (C), and DMFS (D) for pCR patients, who had baseline cN-positive stage, treated with or without ACT (1:3 matching).
Table 1. Patients Baseline Characteristics Before and After Propensity Score Matching

## Slide 2
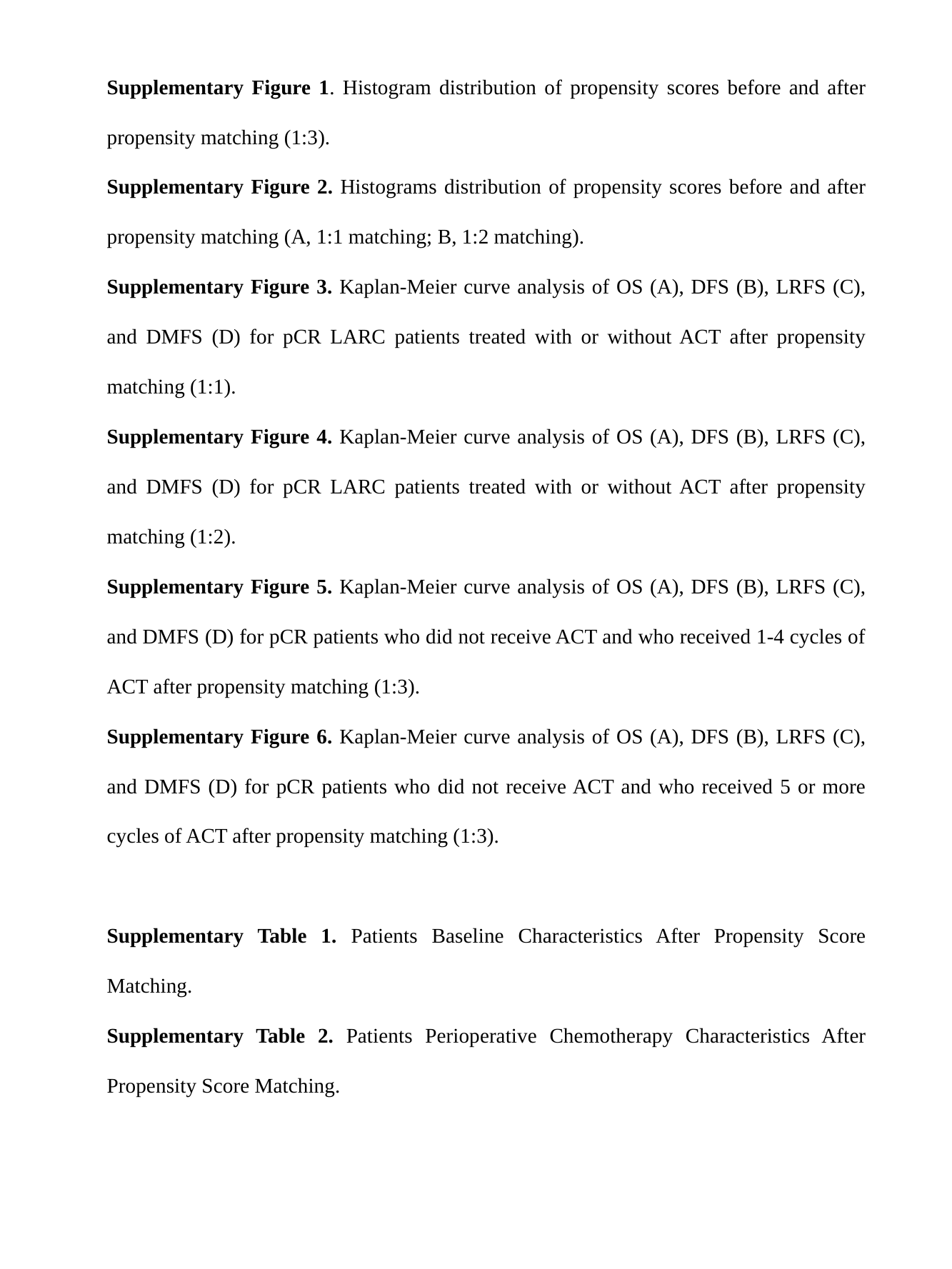

Supplementary Figure 1. Histogram distribution of propensity scores before and after propensity matching (1:3).
Supplementary Figure 2. Histograms distribution of propensity scores before and after propensity matching (A, 1:1 matching; B, 1:2 matching).
Supplementary Figure 3. Kaplan-Meier curve analysis of OS (A), DFS (B), LRFS (C), and DMFS (D) for pCR LARC patients treated with or without ACT after propensity matching (1:1).
Supplementary Figure 4. Kaplan-Meier curve analysis of OS (A), DFS (B), LRFS (C), and DMFS (D) for pCR LARC patients treated with or without ACT after propensity matching (1:2).
Supplementary Figure 5. Kaplan-Meier curve analysis of OS (A), DFS (B), LRFS (C), and DMFS (D) for pCR patients who did not receive ACT and who received 1-4 cycles of ACT after propensity matching (1:3).
Supplementary Figure 6. Kaplan-Meier curve analysis of OS (A), DFS (B), LRFS (C), and DMFS (D) for pCR patients who did not receive ACT and who received 5 or more cycles of ACT after propensity matching (1:3).
Supplementary Table 1. Patients Baseline Characteristics After Propensity Score Matching.
Supplementary Table 2. Patients Perioperative Chemotherapy Characteristics After Propensity Score Matching.

## Slide 3
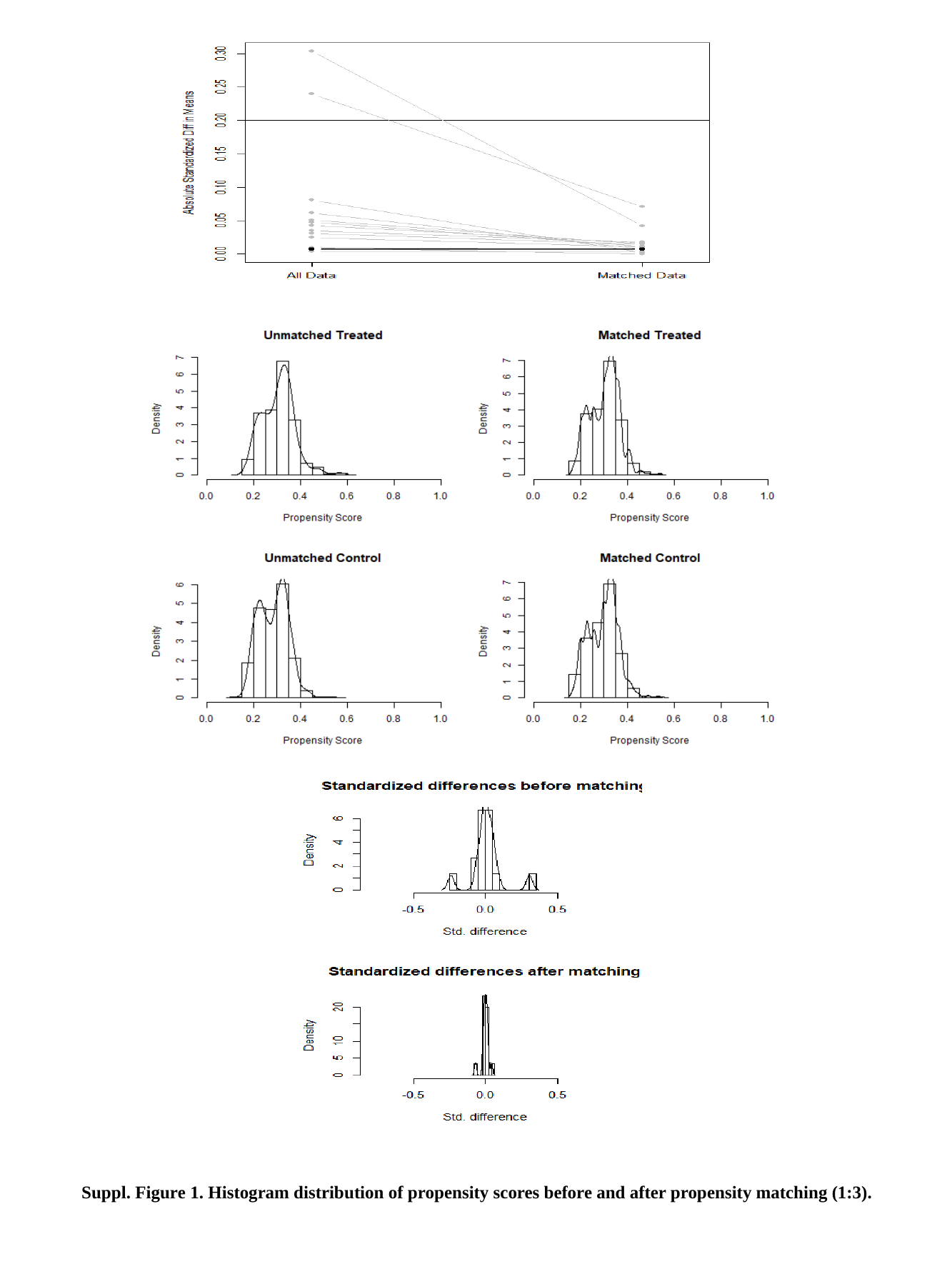

Suppl. Figure 1. Histogram distribution of propensity scores before and after propensity matching (1:3).

## Slide 4
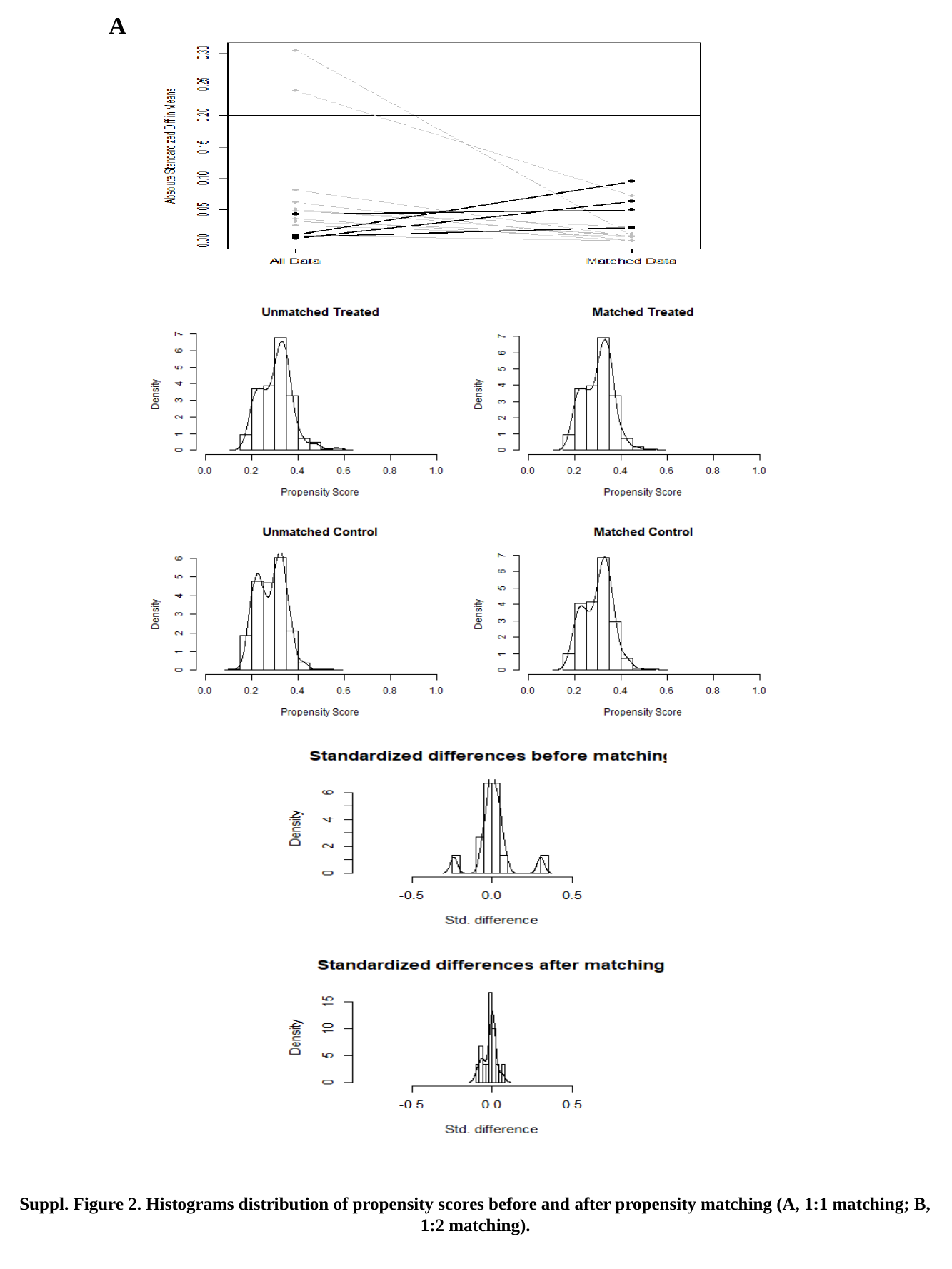

A
Suppl. Figure 2. Histograms distribution of propensity scores before and after propensity matching (A, 1:1 matching; B, 1:2 matching).

## Slide 5
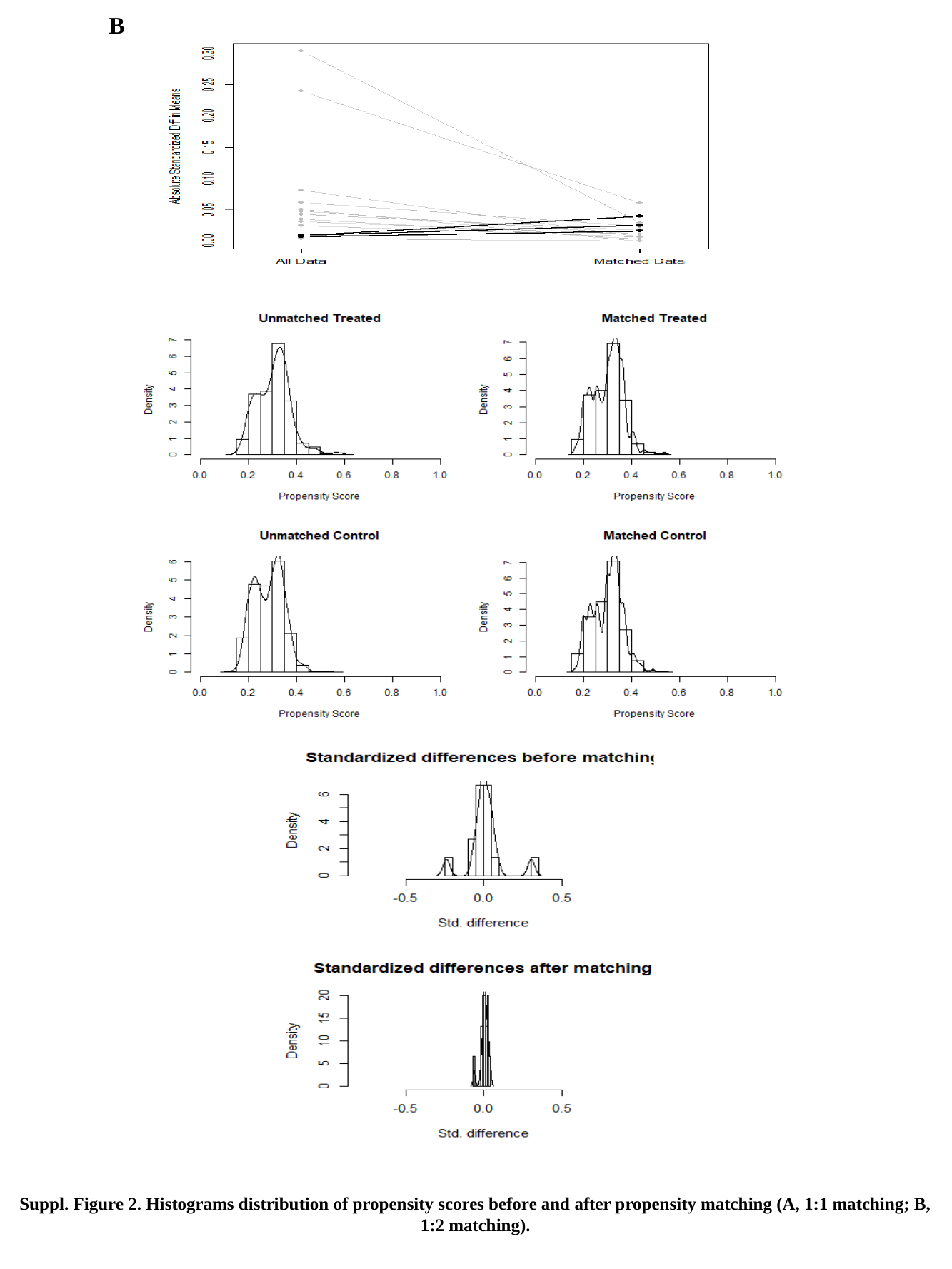

B
Suppl. Figure 2. Histograms distribution of propensity scores before and after propensity matching (A, 1:1 matching; B, 1:2 matching).

## Slide 6
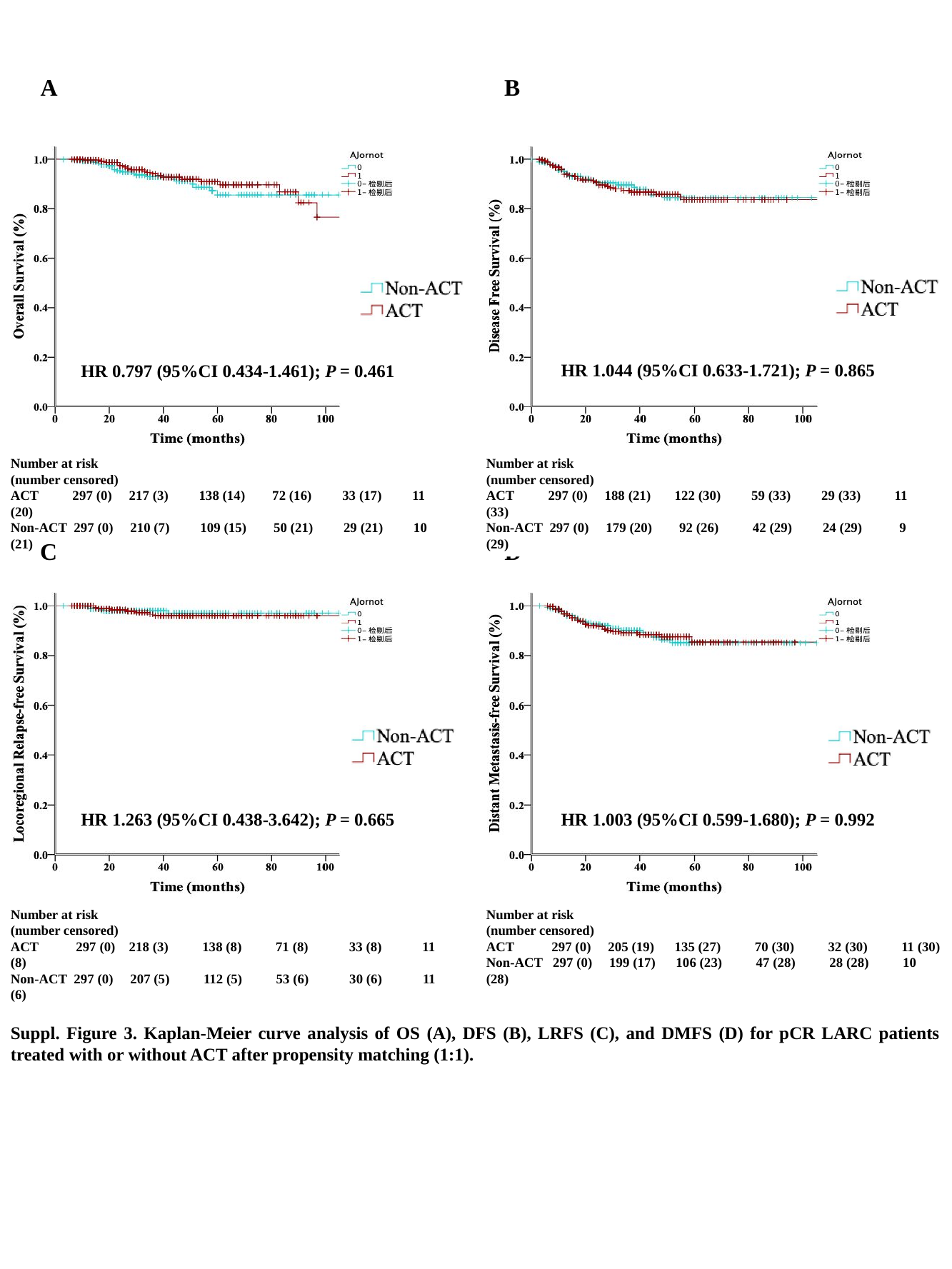

Number at risk
(number censored)
ACT 297 (0) 217 (3) 138 (14) 72 (16) 33 (17) 11 (20)
Non-ACT 297 (0) 210 (7) 109 (15) 50 (21) 29 (21) 10 (21)
A
B
HR 1.044 (95%CI 0.633-1.721); P = 0.865
HR 0.797 (95%CI 0.434-1.461); P = 0.461
Number at risk
(number censored)
ACT 297 (0) 188 (21) 122 (30) 59 (33) 29 (33) 11 (33)
Non-ACT 297 (0) 179 (20) 92 (26) 42 (29) 24 (29) 9 (29)
D
C
HR 1.003 (95%CI 0.599-1.680); P = 0.992
HR 1.263 (95%CI 0.438-3.642); P = 0.665
Number at risk
(number censored)
ACT 297 (0) 218 (3) 138 (8) 71 (8) 33 (8) 11 (8)
Non-ACT 297 (0) 207 (5) 112 (5) 53 (6) 30 (6) 11 (6)
Number at risk
(number censored)
ACT 297 (0) 205 (19) 135 (27) 70 (30) 32 (30) 11 (30)
Non-ACT 297 (0) 199 (17) 106 (23) 47 (28) 28 (28) 10 (28)
Suppl. Figure 3. Kaplan-Meier curve analysis of OS (A), DFS (B), LRFS (C), and DMFS (D) for pCR LARC patients treated with or without ACT after propensity matching (1:1).

## Slide 7
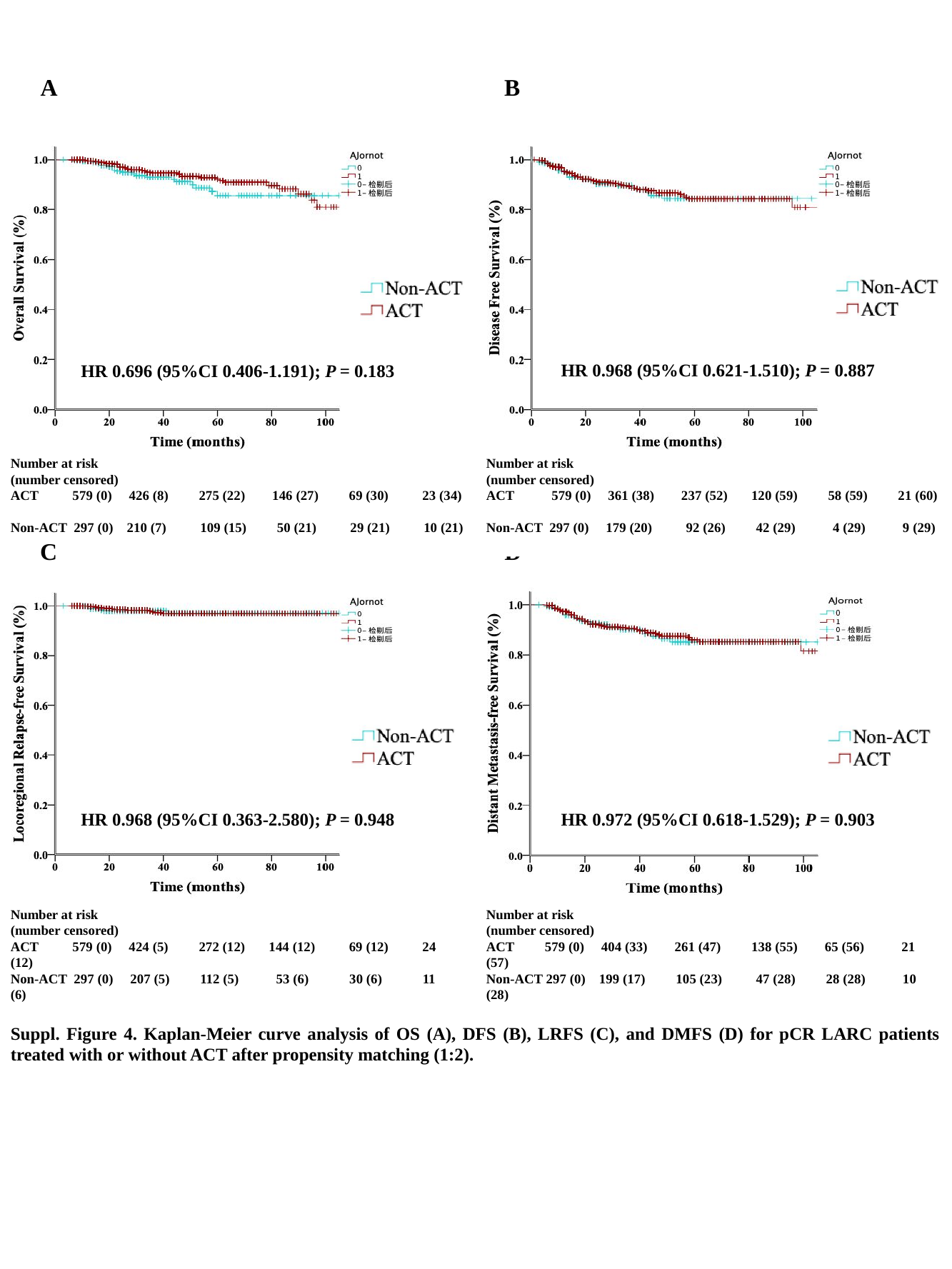

Number at risk
(number censored)
ACT 579 (0) 426 (8) 275 (22) 146 (27) 69 (30) 23 (34)
Non-ACT 297 (0) 210 (7) 109 (15) 50 (21) 29 (21) 10 (21)
A
B
HR 0.968 (95%CI 0.621-1.510); P = 0.887
HR 0.696 (95%CI 0.406-1.191); P = 0.183
Number at risk
(number censored)
ACT 579 (0) 361 (38) 237 (52) 120 (59) 58 (59) 21 (60)
Non-ACT 297 (0) 179 (20) 92 (26) 42 (29) 4 (29) 9 (29)
D
C
HR 0.972 (95%CI 0.618-1.529); P = 0.903
HR 0.968 (95%CI 0.363-2.580); P = 0.948
Number at risk
(number censored)
ACT 579 (0) 424 (5) 272 (12) 144 (12) 69 (12) 24 (12)
Non-ACT 297 (0) 207 (5) 112 (5) 53 (6) 30 (6) 11 (6)
Number at risk
(number censored)
ACT 579 (0) 404 (33) 261 (47) 138 (55) 65 (56) 21 (57)
Non-ACT 297 (0) 199 (17) 105 (23) 47 (28) 28 (28) 10 (28)
Suppl. Figure 4. Kaplan-Meier curve analysis of OS (A), DFS (B), LRFS (C), and DMFS (D) for pCR LARC patients treated with or without ACT after propensity matching (1:2).

## Slide 8
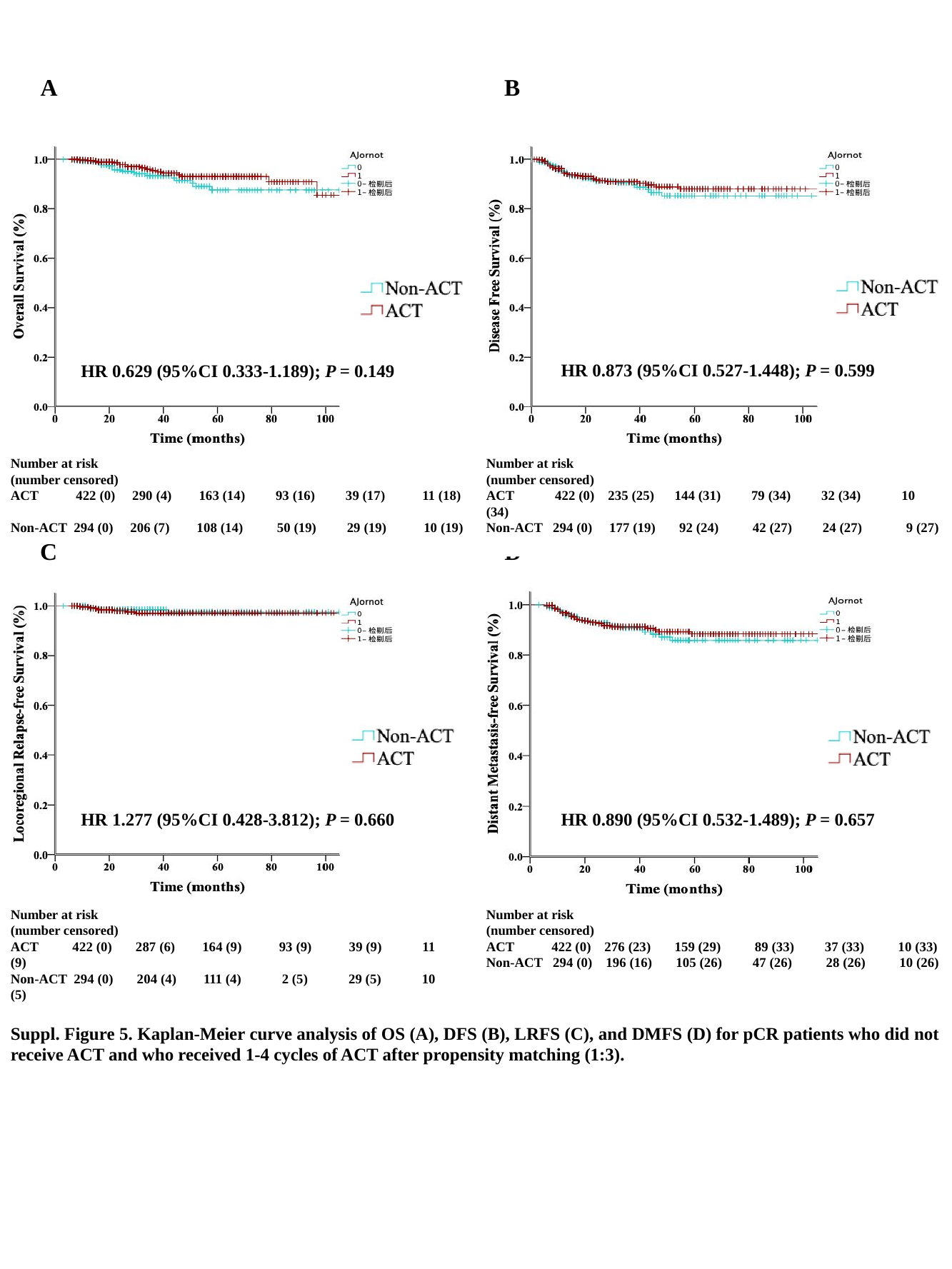

Number at risk
(number censored)
ACT 422 (0) 290 (4) 163 (14) 93 (16) 39 (17) 11 (18)
Non-ACT 294 (0) 206 (7) 108 (14) 50 (19) 29 (19) 10 (19)
A
B
HR 0.873 (95%CI 0.527-1.448); P = 0.599
HR 0.629 (95%CI 0.333-1.189); P = 0.149
Number at risk
(number censored)
ACT 422 (0) 235 (25) 144 (31) 79 (34) 32 (34) 10 (34)
Non-ACT 294 (0) 177 (19) 92 (24) 42 (27) 24 (27) 9 (27)
D
C
HR 0.890 (95%CI 0.532-1.489); P = 0.657
HR 1.277 (95%CI 0.428-3.812); P = 0.660
Number at risk
(number censored)
ACT 422 (0) 287 (6) 164 (9) 93 (9) 39 (9) 11 (9)
Non-ACT 294 (0) 204 (4) 111 (4) 2 (5) 29 (5) 10 (5)
Number at risk
(number censored)
ACT 422 (0) 276 (23) 159 (29) 89 (33) 37 (33) 10 (33)
Non-ACT 294 (0) 196 (16) 105 (26) 47 (26) 28 (26) 10 (26)
Suppl. Figure 5. Kaplan-Meier curve analysis of OS (A), DFS (B), LRFS (C), and DMFS (D) for pCR patients who did not receive ACT and who received 1-4 cycles of ACT after propensity matching (1:3).

## Slide 9
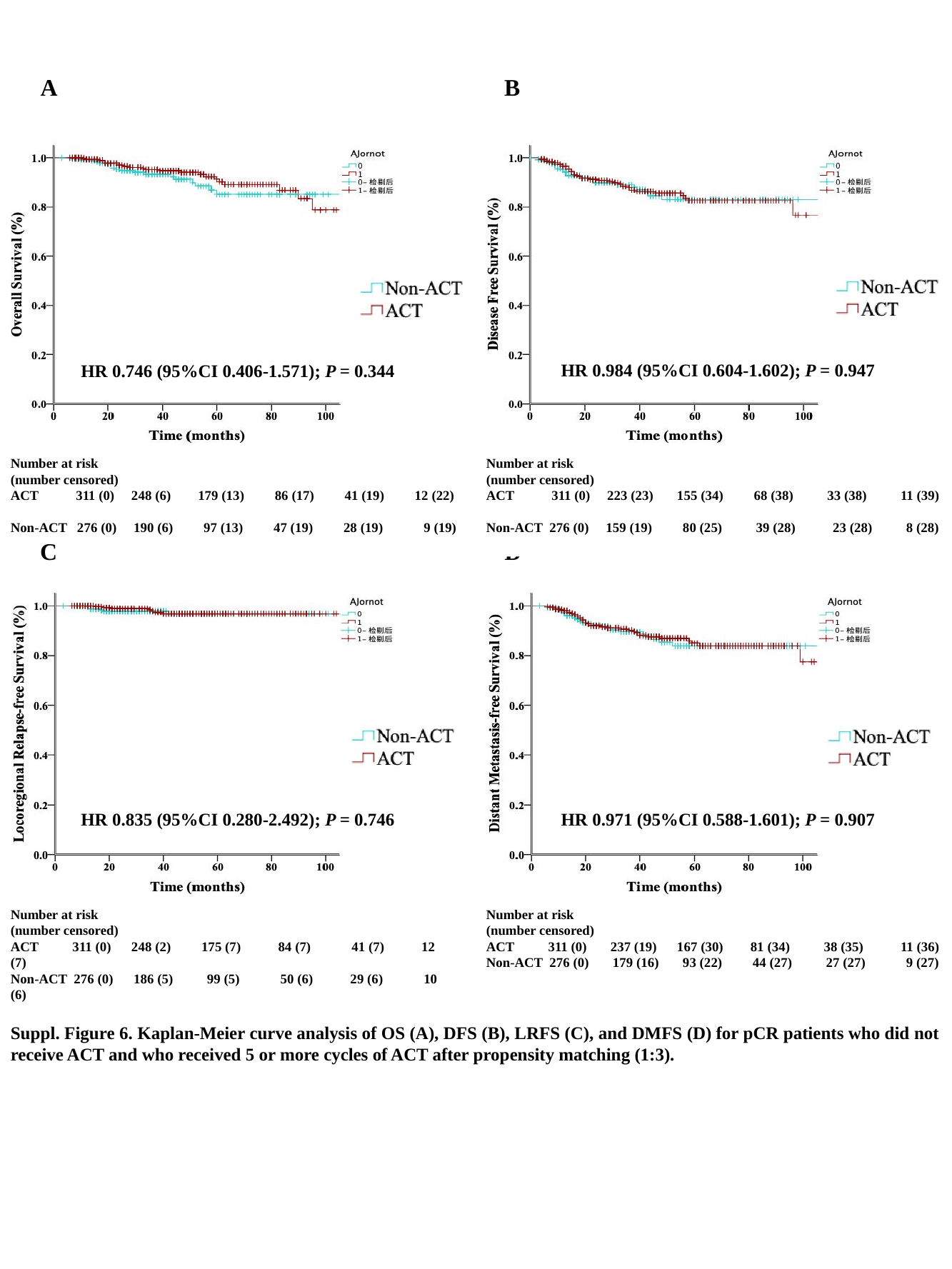

Number at risk
(number censored)
ACT 311 (0) 248 (6) 179 (13) 86 (17) 41 (19) 12 (22)
Non-ACT 276 (0) 190 (6) 97 (13) 47 (19) 28 (19) 9 (19)
A
B
HR 0.984 (95%CI 0.604-1.602); P = 0.947
HR 0.746 (95%CI 0.406-1.571); P = 0.344
Number at risk
(number censored)
ACT 311 (0) 223 (23) 155 (34) 68 (38) 33 (38) 11 (39)
Non-ACT 276 (0) 159 (19) 80 (25) 39 (28) 23 (28) 8 (28)
D
C
HR 0.971 (95%CI 0.588-1.601); P = 0.907
HR 0.835 (95%CI 0.280-2.492); P = 0.746
Number at risk
(number censored)
ACT 311 (0) 248 (2) 175 (7) 84 (7) 41 (7) 12 (7)
Non-ACT 276 (0) 186 (5) 99 (5) 50 (6) 29 (6) 10 (6)
Number at risk
(number censored)
ACT 311 (0) 237 (19) 167 (30) 81 (34) 38 (35) 11 (36)
Non-ACT 276 (0) 179 (16) 93 (22) 44 (27) 27 (27) 9 (27)
Suppl. Figure 6. Kaplan-Meier curve analysis of OS (A), DFS (B), LRFS (C), and DMFS (D) for pCR patients who did not receive ACT and who received 5 or more cycles of ACT after propensity matching (1:3).

## Slide 10
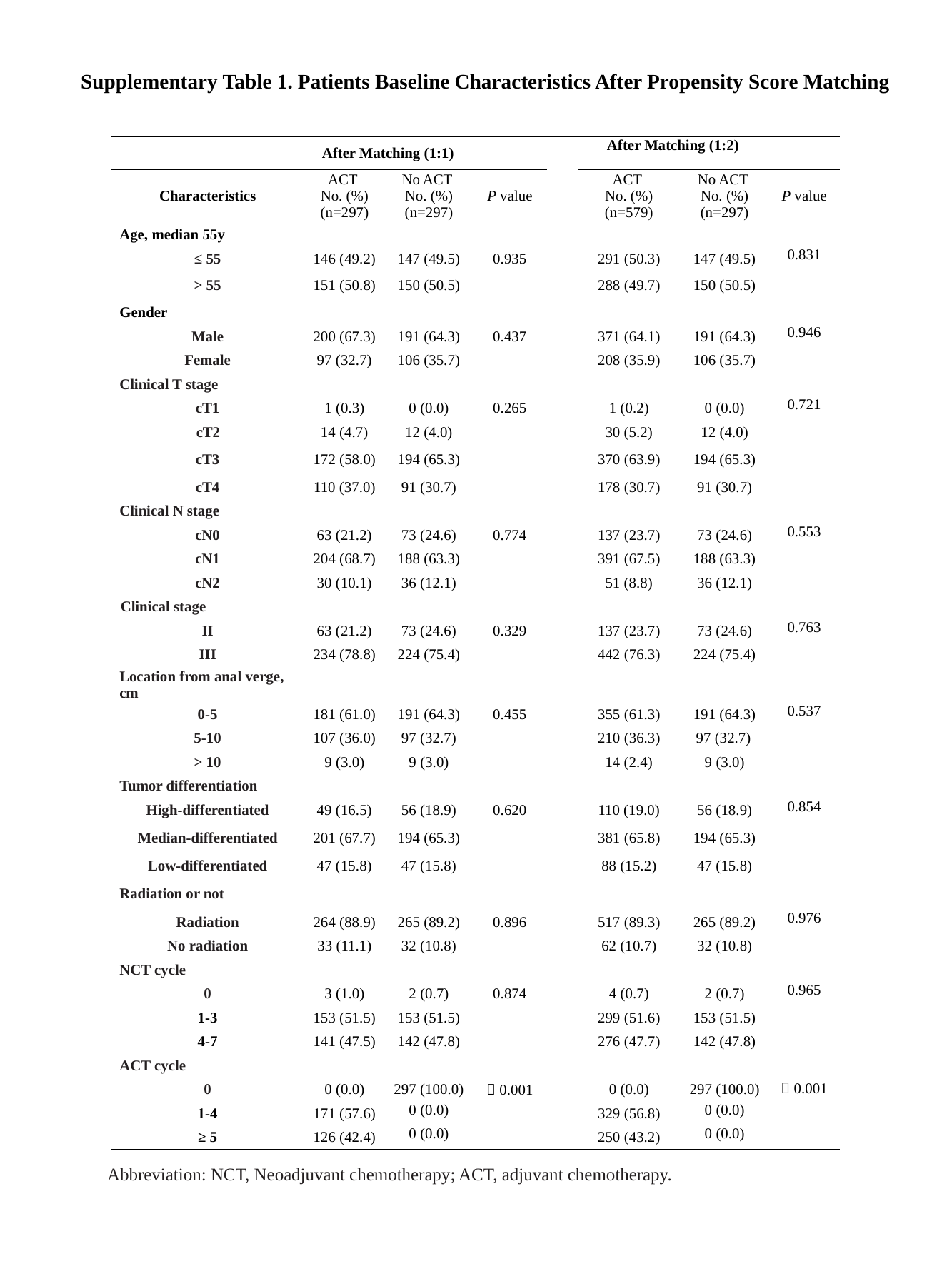

Supplementary Table 1. Patients Baseline Characteristics After Propensity Score Matching
| | After Matching (1:1) | | | | After Matching (1:2) | | |
| --- | --- | --- | --- | --- | --- | --- | --- |
| Characteristics | ACT No. (%) (n=297) | No ACT No. (%) (n=297) | P value | | ACT No. (%) (n=579) | No ACT No. (%) (n=297) | P value |
| Age, median 55y | | | | | | | |
| ≤ 55 | 146 (49.2) | 147 (49.5) | 0.935 | | 291 (50.3) | 147 (49.5) | 0.831 |
| > 55 | 151 (50.8) | 150 (50.5) | | | 288 (49.7) | 150 (50.5) | |
| Gender | | | | | | | |
| Male | 200 (67.3) | 191 (64.3) | 0.437 | | 371 (64.1) | 191 (64.3) | 0.946 |
| Female | 97 (32.7) | 106 (35.7) | | | 208 (35.9) | 106 (35.7) | |
| Clinical T stage | | | | | | | |
| cT1 | 1 (0.3) | 0 (0.0) | 0.265 | | 1 (0.2) | 0 (0.0) | 0.721 |
| cT2 | 14 (4.7) | 12 (4.0) | | | 30 (5.2) | 12 (4.0) | |
| cT3 | 172 (58.0) | 194 (65.3) | | | 370 (63.9) | 194 (65.3) | |
| cT4 | 110 (37.0) | 91 (30.7) | | | 178 (30.7) | 91 (30.7) | |
| Clinical N stage | | | | | | | |
| cN0 | 63 (21.2) | 73 (24.6) | 0.774 | | 137 (23.7) | 73 (24.6) | 0.553 |
| cN1 | 204 (68.7) | 188 (63.3) | | | 391 (67.5) | 188 (63.3) | |
| cN2 | 30 (10.1) | 36 (12.1) | | | 51 (8.8) | 36 (12.1) | |
| Clinical stage | | | | | | | |
| II | 63 (21.2) | 73 (24.6) | 0.329 | | 137 (23.7) | 73 (24.6) | 0.763 |
| III | 234 (78.8) | 224 (75.4) | | | 442 (76.3) | 224 (75.4) | |
| Location from anal verge, cm | | | | | | | |
| 0-5 | 181 (61.0) | 191 (64.3) | 0.455 | | 355 (61.3) | 191 (64.3) | 0.537 |
| 5-10 | 107 (36.0) | 97 (32.7) | | | 210 (36.3) | 97 (32.7) | |
| > 10 | 9 (3.0) | 9 (3.0) | | | 14 (2.4) | 9 (3.0) | |
| Tumor differentiation | | | | | | | |
| High-differentiated | 49 (16.5) | 56 (18.9) | 0.620 | | 110 (19.0) | 56 (18.9) | 0.854 |
| Median-differentiated | 201 (67.7) | 194 (65.3) | | | 381 (65.8) | 194 (65.3) | |
| Low-differentiated | 47 (15.8) | 47 (15.8) | | | 88 (15.2) | 47 (15.8) | |
| Radiation or not | | | | | | | |
| Radiation | 264 (88.9) | 265 (89.2) | 0.896 | | 517 (89.3) | 265 (89.2) | 0.976 |
| No radiation | 33 (11.1) | 32 (10.8) | | | 62 (10.7) | 32 (10.8) | |
| NCT cycle | | | | | | | |
| 0 | 3 (1.0) | 2 (0.7) | 0.874 | | 4 (0.7) | 2 (0.7) | 0.965 |
| 1-3 | 153 (51.5) | 153 (51.5) | | | 299 (51.6) | 153 (51.5) | |
| 4-7 | 141 (47.5) | 142 (47.8) | | | 276 (47.7) | 142 (47.8) | |
| ACT cycle | | | | | | | |
| 0 | 0 (0.0) | 297 (100.0) | ＜0.001 | | 0 (0.0) | 297 (100.0) | ＜0.001 |
| 1-4 | 171 (57.6) | 0 (0.0) | | | 329 (56.8) | 0 (0.0) | |
| ≥ 5 | 126 (42.4) | 0 (0.0) | | | 250 (43.2) | 0 (0.0) | |
Abbreviation: NCT, Neoadjuvant chemotherapy; ACT, adjuvant chemotherapy.

## Slide 11
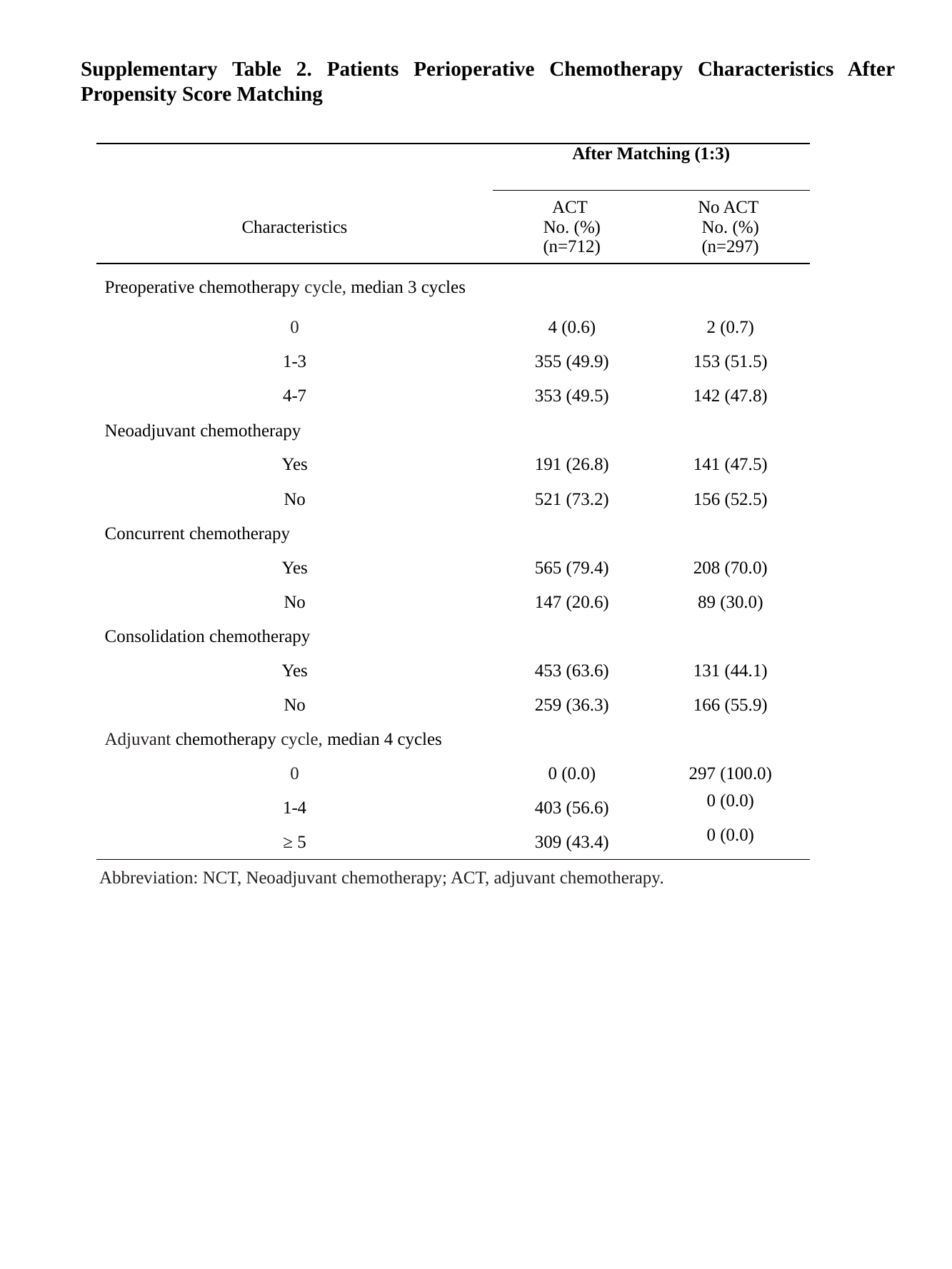

Supplementary Table 2. Patients Perioperative Chemotherapy Characteristics After Propensity Score Matching
| | After Matching (1:3) | |
| --- | --- | --- |
| Characteristics | ACT No. (%) (n=712) | No ACT No. (%) (n=297) |
| Preoperative chemotherapy cycle, median 3 cycles | | |
| 0 | 4 (0.6) | 2 (0.7) |
| 1-3 | 355 (49.9) | 153 (51.5) |
| 4-7 | 353 (49.5) | 142 (47.8) |
| Neoadjuvant chemotherapy | | |
| Yes | 191 (26.8) | 141 (47.5) |
| No | 521 (73.2) | 156 (52.5) |
| Concurrent chemotherapy | | |
| Yes | 565 (79.4) | 208 (70.0) |
| No | 147 (20.6) | 89 (30.0) |
| Consolidation chemotherapy | | |
| Yes | 453 (63.6) | 131 (44.1) |
| No | 259 (36.3) | 166 (55.9) |
| Adjuvant chemotherapy cycle, median 4 cycles | | |
| 0 | 0 (0.0) | 297 (100.0) |
| 1-4 | 403 (56.6) | 0 (0.0) |
| ≥ 5 | 309 (43.4) | 0 (0.0) |
Abbreviation: NCT, Neoadjuvant chemotherapy; ACT, adjuvant chemotherapy.
